# The immunomodulatory effects of sodium new houttuyfonate on different states of macrophage against *Aspergillus fumigatus* infection via distinct mechanism in invasive pulmonary aspergillosis

**DOI:** 10.1186/s13020-025-01157-3

**Published:** 2025-07-02

**Authors:** Xue Zhou, Fujiao Huang, Jinping Zhang, Shu Gong, Liwen Wei, Fangyang Liu, Zhangyong Song

**Affiliations:** 1 School of Basic Medical Sciences, Southwest Medical University, Luzhou, 646000 People’s Republic of China; 2 Tuberculosis Referrence Laboratory, Chongqing Municipal Institute of Tuberculosis, Chongqing, 400050 China; 3 Public Center of Experimental Technology, Southwest Medical University, Luzhou, 646000 People’s Republic of China; 4 Hemodynamics and Medical Engineering Combination Key Laboratory of Luzhou, Luzhou, 646000 China

**Keywords:** Sodium new houttuyfonate, microRNA, Invasive pulmonary aspergillosis, Macrophage, *Aspergillus fumigatus*

## Abstract

**Background:**

Invasive pulmonary aspergillosis (IPA) is usually caused by *Aspergillus fumigatus* infection and associated with high morbidity and mortality in immunocompromised patients. Sodium new houttuyfonate (SNH) has antifungal and immunomodulatory properties, but the therapeutic effect against IPA remains unknown.

**Methods:**

The therapeutic effect of SNH in mice with IPA was determined by histopathological analysis and measurements of fungal loads. The toxicity of SNH to mouse alveolar macrophages (MH-S cells) and the changes in phagocytosis ability of MH-S cells to *A. fumigatus* were determined by cell counting kit-8 (CCK8), microscopic observation, respectively. The biological mechanism of SNH regulating the functional changes of MH-S cells was determined by transcriptomics, real-time quantitative polymerase chain reaction (RT-qPCR), western blot, molecular docking, microRNA sequencing, and miRNA_mRNA interaction analyses.

**Results:**

Intraperitoneal injection of SNH had a therapeutic effect in mice with IPA, and 8 μg/mL of SNH was significantly enhanced the ability of MH-S cells to phagocytize and kill *A. fumigatus* conidia. Moreover, SNH increased antifungal activity of MH-S cells to *A. fumigatus* via the TLR2/TRAF6/IRF5 and TLR2/TRAF6/ERK axes, respectively. What’s more, SNH inhibited the expression of miR-328-5p and miR-6975-3p in MH-S cells, which negatively regulated TLR2 expression and the phagocytosis and killing functions of MH-S cells.

**Conclusions:**

Taken together, these findings clarify the potential immunomodulatory mechanism of SNH for treatment of IPA, suggesting that SNH is effective against *A. fumigatus* infection.

## Background

Typically, hundreds of *Aspergillus fumigatus* conidia enter the body daily through the respiratory tract [1]. In healthy individuals with normal immune function, *A. fumigatus* conidia are cleared by the respiratory mucociliary system, alveolar macrophages (AMs), and other immune cells, while conidia germinate and form invasive mycelia leading to persistent lung disease in immunocompromised individuals [2]. It found that about one-third of the 3.23 million deaths annually from chronic obstructive pulmonary disease worldwide are directly related to *Aspergillus* infections [3]. Deaths due to *Aspergillus* infections are secondary to influenza virus and the mortality rate of influenza-associated aspergillus disease is reportedly up to 100% [4]. Invasive aspergillosis, allergic pulmonary aspergillosis, and chronic pulmonary obstructive pulmonary disease are the three most common diseases caused by *A*. *fumigatus*, of which the fatality rate of invasive aspergillosis is > 50% [2, 5]. Invasive pulmonary aspergillosis (IPA) is the most common and severe form of invasive aspergillosis. Moreover, the incidence of IPA has increased along with the development of advanced therapies, such as organ transplantation [6, 7].

AMs are resident macrophages of the lung derived from monocytic cells and account for > 90% of lung leukocytes [8]. As the first line of defense in the lungs, AMs play important roles in innate immunity by recognition, processing, and presenting of antigens to induce specific immune responses. Moreover, AMs maintain homeostasis of the local microenvironment and prevent excessive immune damage through negative feedback [9, 10]. Also, AMs recognize and phagocytize the conidia of *A. fumigatus* in the lungs and regulate cytokine production. The recognition of *A. fumigatus* conidia by AMs is dependent on pathogen pattern recognition receptors (PRR), such as toll-like receptors (TLRs), nucleotide binding oligomerization domain protein family members, and C-type lectin receptors [11]. As essential components of the innate immune system, TLRs play an important role in detection of specific microbe-associated molecular patterns [12]. TLR2 is an important membrane receptor that mainly mediates recognition of various cell wall components of *Aspergillus*, such as α(1,3)- and β(1,3)-glucans, chitin, and galactomannans [13, 14]. Reactive oxygen species (ROS) produced by neutrophils and the formation of extracellular traps are indispensable for clearance of *A. fumigatus* [15]. In addition, the presence or absence of T cells is closely related to the prognosis of *A. fumigatus* infection. T cells release perforin and granzyme B, which confer direct antifungal effects [16]. Meanwhile, clinical studies have shown that production of specific T cells is positively correlated with a good prognosis of *A. fumigatus* infection, although the factors that induce differentiation of specific T cells remain unknown [17, 18]. The activation of neutrophils and T cells was closely related to the different states of macrophages [19], however, how Sodium new houttuyfonate (SNH) affects the immune status of AMs is unclear.

SNH is a modified compound derived from the traditional Chinese herb Houttuynia *cordata Thunb* [20], which is commonly used for treatment of purulent skin infections and respiratory infections [21–23]. SNH is widely used in the pharmaceutical and medical fields for treatment of fungal and bacterial infections, and control tumor growth [24]. For example, SNH has been shown to enhance phagocytosis and killing of *Pseudomonas aeruginosa* through the TLR4/nuclear factor kappa-B (NF-κB) pathway in mouse macrophages (RAW264.7 cells) [25]. Moreover, SNH was reported to inhibit growth of non-small cell lung cancer cells by pyroptosis through the TCON-14036/miR-1228-5p/PRKCDBP pathway [21], and suppress metastasis through the Linc00668/miR-147a/Slug axis [26]. In addition, a previous study by our group confirmed that SNH had antifungal *A. fumigatus* activity and could reduce the fungal load in the liver and kidney tissues in mice with disseminated *A. fumigatus* infection [27]. However, the specific immune regulatory mechanism in response to *A. fumigatus* infection remains unclear.

MicroRNAs (miRNAs) are a class of non-coding single-stranded RNAs with a length of 19–25 nucleotides that play important roles in post-transcriptional regulation of gene expression [28]. Previous investigations have linked miRNAs with the occurrence and development of asthma, eosinophilic esophagitis, allergic rhinitis, eczema, and cancer [29–31]. Also, miRNAs may play an important role in the regulation of macrophage polarization. For example, miR-181a, miR-155-5p, miR-204-5p, and miR-451 are significantly up-regulated in M1-polarized macrophages, while miR-181A, miR-155-5p, and miR-451 are significantly up-regulated, and miR-125-5p, miR-146a-3p, miR-143-3p, and miR-145-5p are significantly down-regulated in M2-polarized cells [32]. In addition, SNH has been confirmed to regulate expression of miR-147a for treatment of lung cancer [26]. Therefore, targeting miRNAs is an emerging therapeutic approach.

Therefore, the aim of the present study was to clarify the therapeutic effect and the immunomodulatory mechanism of SNH against *A. fumigatus* infection in a mouse model of IPA. The results showed that SNH had a therapeutic effect against *A*. *fumigatus* infection and improved the ability of MH-S cells to phagocytize and kill *A. fumigatus* conidia. Moreover, SNH down-regulated expression of miR-328-5p and miR-6975-3p that negatively regulated the TLR2/TRAF6/IRF5 and TLR2/TRAF6/ERK1 axes, respectively, and regulated the expression of genes coding for cytokines and chemokines in MH-S cells. These results suggest that SNH not only has an antifungal activity, but also has immunomodulatory effect for treatment of *A. fumigatus* infection.

## Methods

### Strain, cell lines, media, drugs, and growth conditions

Before the experiment, the standard *A. fumigatus* strain AF293 was cultured on potato dextrose agar (PDA) medium at 37 °C for 3–7 days as described previously [33]. MH-S cells were cultured in complete Dulbecco’s modified Eagle’s medium (Thermo Fisher Scientific, Waltham, MA, USA) containing 10% fetal bovine serum (Jiangsu MRC Biological Technology Co., Ltd., Jiangsu, China) and 1% penicillin–streptomycin (100 ×) (Beyotime Institute of Biotechnology, Shanghai, China) at 37 °C under an atmosphere of 5% CO_2_. SNH (purity ≥ 98%) was purchased from Source Leaf Biotech Company (Shanghai, China).

### Therapeutic effect of SNH against IPA

Female C57 mice (n = 60; age, 6–8 weeks; body weight, 20–25 g; Laboratory Animal Center, Southwest Medical University, Luzhou, Sichuan) were randomly assigned to one of six groups: an untreated negative control (blank) group, an immunosuppressed untreated (control) group, or one of four experimental groups of immunosuppressed mice exposed to *A. fumigatus* and either untreated (model group) or treated with itraconazole at 75 mg/kg/day or SNH at 10 or 30 mg/kg/day [34, 35]. To induce immunosuppression, the mice were subcutaneously injected with cortisone acetate at 225 mg/kg on days − 2 and 0. Except for the blank and control groups, mice in the other groups received a nasal drip of *A*. *fumigatus* conidia suspension (50 μL; 1 × 10^8^ conidia/mL) for 3 consecutive days. After successful establishment of IPA, the mice were intraperitoneally injected with of SNH at 10 or 30 mg/kg/day for 3 and 5 days.

The effect of SNH against *A*. *fumigatus* infection was determined by measuring the fungal burden and histopathological analyses. The lungs were collected after 3 and 5 days of treatment, weighed, homogenized, then smeared on plates and incubated at 37 ℃ for 36 h. Then, the colony-forming units (CFUs) were counted [36]. After euthanasia, the mice underwent cardiac perfusion. The whole lung tissues were fixed with 4% methanol, embedded in paraffin, and cut into thin slices, which were stained with hematoxylin–eosin (H&E) and periodic acid-schiff (PAS) stain [37].

The inflammatory status and recruitment of immune cells were evaluated using pulmonary immune cell assays [38]. Antibodies against immune cells were purchased from Shanghai Universal Biotech Co., Ltd. (Shanghai, China). After euthanasia, the lung tissues were resected and digested in Dulbecco’s modified Eagle’s medium containing 5% fetal bovine serum and type I collagenase (Solarbio, Beijing, China) for 1 h. Then, the cells were collected and incubated with rat anti-mouse antibodies against CD16/CD32 for 10–20 min at room temperature, followed by premixed antibodies against CD45, CD11b, Ly-6G, CD3e, and F4/80 at room temperature for 15 min in the dark. After washing with phosphate-buffered saline (PBS), the cells were incubated with 4′,6-diamidino-2-phenylindole (5 μL) for 5 min and resuspended in 300 μL of PBS prior to flow cytometry (POWCLIN, Hangzhou, China).

### Cell viability and morphological change assay

The effect of SNH on the viability of MH-S cells was measured with a cell counting kit-8 (CCK-8) assay (Apexbio Technology LLC, Houston, TX, USA), as described previously [39]. Briefly, MH-S cells were inoculated at 2 × 10^5^ cells/mL into the wells of 96-well plates and cultured overnight. Then, the culture was continued with various concentrations of SNH at 0–100 μg/mL (increments of 10 μg) for 2 h and 0–20 μg/mL (increments of 2 μg) for 4 h, respectively. Afterward, 10 μL of CCK-8 reagent were added to each well and incubation was continued at 37 °C for 2 h. Absorbance of a multifunctional enzyme marker (Thermo Fisher Scientific, Waltham, MA, USA) was measured at 490 nm. Each experiment was independently repeated three times.

To confirm the morphological effects of SNH, MH-S cells were incubated with or without *A. fumigatus* conidia (1 conidia per cell) at 37 ℃ for 2 h under an atmosphere of 5% CO_2_, followed by overnight culture in the wells of 12-well plates (1 × 10^5^ cells/mL) with round coverslips. Then, SNH was added to the wells containing MH-S cells cultured with and without *A. fumigatus* conidia and incubation was continued for 2 or 4 h. Afterward, the cells were observed under a microscope (Olympus Corporation, Tokyo, Japan). This experiment was independently repeated three times.

### Phagocytosis and killing ability of conidia

A phagocytosis assay was performed in accordance with an established method with slight modifications [40]. Briefly, MH-S cells (2 mL, 2 × 10^5^ cells/mL) were added to the wells of a six-well plate with round coverslips. After culturing overnight, the MH-S cells were treated with SNH (0, 4, 8, 12, or 16 μg/mL) for 2 or 4 h. After removal of the SNH supernatant, the MH-S cells were co-cultured for 2 h with a spore suspension (5 conidia per cell) and then rinsed three times with pre-cooled PBS at 4 °C to stop phagocytosis and remove non-phagocytosed conidia. Finally, the extracellular conidia were labeled with calcofluor white stain (Solarbio, Beijing, China) [41], and the rate of phagocytosis was observed under a microscope (Olympus Corporation) to determine the proportion of phagocytized conidia. In total, 200 MH-S cells were randomly counted. The experiment was independently repeated three times. The phagocytosis rate was calculated as the number of MH-S cells containing phagocytized conidia/200 × 100%.

A modified killing assay was performed as described previously [42]. Briefly, cells in suspension (1 mL, 1 × 10^5^ cells/mL) were added to the wells of a 12-well plate and cultured overnight, followed by co-culturing with SNH (0, 4, 8, 12, or 16 μg/mL) for 2 or 4 h. After discarding the supernatant, a spore suspension was added (5 conidia per cell) and the culture was continued for 2 h. After rinsing with precooled PBS to stop phagocytosis and remove the non-phagocytized conidia, half of the cells were cultured for an additional 6 h. The remaining cells were placed in ice-cold sterilized water to induce lysis and release the phagocytized conidia. The cell lysates were collected in individual tubes, serially diluted, and cultured on PDA for 36 h at 37 °C. Then, the CFUs were counted. This assay was independently repeated three times. The killing rate was calculated as 1—(number of CFUs after culture with SNH for an addition 6 h/number of CFUs after co-culture without SNH for an additional 6 h) × 100%.

### Production of ROS

To confirm ROS production, MH-S cells (1 × 10^6^ cells/mL) were added to the wells of a 6-well plate and cultured with 8 μg/mL of SNH for 0, 1, 2, 3, or 4 h. Following the addition of 2, 7-dichlorofluorescein diacetate (10 μM/mL; Merck & Co Inc., Rahway, NJ, USA), the treated cells were incubated at 37 °C for 30 min [43]. Afterward, the cells were resuspended in PBS for flow cytometry, which was conducted with a BD FACSCalibur™ Flow Cytometer (BD Biosciences, San Jose, CA, USA) and analyzed with FlowJo^™^ software version 10.8.1. For ROS imaging, after treatment and staining as described above, the samples were washed with pre-cooled PBS and then photographed with a fluorescence microscope (Olympus Corporation). This assay was independently repeated three times.

### Molecular docking, transcriptomic analysis, miRNA sequencing, and Real-time quantitative polymerase chain reaction (RT-qPCR) assay

To clarify the effect of SNH on the ability of MH-S cells to phagocytize and kill conidia, MH-S cells (1 × 10^6^ cells/mL) cultured with or without *A. fumigatus* conidia were incubated with SNH (8 μg/mL) in the wells of a 6-well plate for 1 h. Total RNA was collected and sequenced by Biomarker Technologies (Qingdao, China). Then, quality control detection of the raw sequence data was performed on the Biomarker platform (www.biocloud.net). Sequencing alignment was performed using the Hisat2 alignment program (version 2.0.4 https://daehwankimlab.github.io/hisat2/). The samtools suite (version 1.9; https://www.htslib.org/doc/1.9/samtools.html) was used to convert the sam files to bam files. Then, the matched reads were assembled and quantified using the StringTie tool (version v2.2.1 https://bioinformaticshome.com/tools/rna-seq/descriptions/StringTie). Differentially expressed genes (DEGs; adjusted *p*-value < 0.01 and fold change ≥ 2) were identified using the DESeq2 software package (version 1.30.1; https://bioconductor.org/packages/release/bioc/html/DESeq2.html). Gene function annotation was performed in reference to the Kyoto Encyclopedia of Genes and Genomes (KEGG) and Gene Ontology (GO) databases. Raw sequence data were deposited in the Genome Sequence Archive of the Beijing Institute of Genomics (accession no. CRA015239).

The miRNA sequences were processed in reference to the transcriptome. Differential expression analysis was performed using the R package DESeq2 (version 1.10.1; https://bioconductor.org/packages/release/bioc/html/DESeq2.html). The resulting *p* values were adjusted using the Benjamini and Hochberg procedure to control the false discovery rate. All miRNAs with |log2(fold change)|≥ 0.58 and *p-*value ≤ 0.05 identified by DESeq2 were considered differentially expressed. Raw sequence data were deposited in the Genome Sequence Archive of the Beijing Institute of Genomics (accession no. CRA015645). The online platform SRplot for data visualization and graphing (http://www.bioinformatics.com.cn) was used to identify associations between the miRNAs and target genes.

The docking targets of SNH and TLR2 were confirmed using the TLR1-TLR2 heterodimer mouse receptor (Protein Data Bank [https://www.rcsb.org/] identification no. 2z81). ChimeraX software (https://www.cgl.ucsf.edu/chimerax/) was used to remove water molecules and bind the lipid in the pocket, set the lipid in the structure as the center, and fill the missing side chain for docking [44]. Moreover, the docking box size was 25 × 25 × 25 and the gnina program (https://github.com/gnina/gnina) was used for docking analysis.

The time-specific expression patterns of miRNAs and genes were determined by RT-qPCR analysis [45]. Briefly, MH-S cells (1 × 10^6^ cells/mL) cultured with or without *A. fumigatus* conidia were incubated with SNH (8 μg/mL) in the wells of 6-well plates for 0, 1, 2, 3, or 4 h. Total RNA was isolated from MH-S cells using TRIzol reagent [46] and reverse transcribed into complementary DNA using PrimeScript RT Master Mix and the Mir-X^™^ miRNA First-Strand Synthesis Kit (Takara Bio, Inc., Shiga, Japan) in accordance with the manufacturer’s instructions. Finally, the miRNA and mRNA expression levels were measured using the 2^−ΔΔCT^ method with U6 miRNA and glyceraldehyde-3-phosphate-dehydrogenase (GAPDH) mRNA as internal controls, respectively [45, 47]. The primer sequences are listed in Supplemental Table 1.

**Table 1:** Top 10 miRNA with the number of regulatory target genes in MH-S cells co-cultureed with SNH

MicroRNAs	Target genes	Up/Down regulation (fold)
miR-328-5p	2024	− 2.560
miR-1230	1135	− 2.910
miR-364	512	4.540
miR-949	287	− 5.377
miR-525	286	− 5.375
miR-3473b	491	− 1.328
miR-3470b	471	− 1.119
miR-52	376	− 1.567
miR-677-3p	284	− 1.969
miR-630	273	− 1.878

### Western blot (WB) analysis

Total protein was isolated from MH-S cells using radioimmunoprecipitation lysis buffer (EpiZyme, Shanghai, China) [48]. The total protein concentration was determined with a bicinchoninic acid assay kit (Beyotime Institute of Biotechnology). Equal amounts of protein were separated by 10% sodium dodecyl sulfate–polyacrylamide gel electrophoresis (EpiZyme) and electroblotted onto polyvinylidene difluoride membranes (EMD Millipore Corporation, Billerica, MA, USA), which were blocked with protein-free rapid blocking solution (EpiZyme) and probed overnight at 4 °C with primary antibodies against TLR2 (ab209217), (dilution, 1:1000; Abcam, Cambridge, UK), TRAF6 (bs-2830R), IRF5 (bs-16703R), ERK (bsm-33232 M), and β-actin (bs-0061R) (dilution, 1:1000; Bioss Antibodies, Beijing, China), then washed three times with Tris-buffered saline/Tween-20 (1 ×), incubated for 2 h with corresponding secondary antibodies (dilution, 1:3000; Beyotime Institute of Biotechnology), and imaged with a Tanon^™^ 5200CE Chemi-Image System (ABclonal Technology, Woburn, MA, USA). This assay was independently repeated three times.

### CRISPR-Cas 13d design strategy

Plasmid construction and cell transfection were performed as described previously with slight modifications [49]. Briefly, target single-guide RNA was designed using an online design tool (https://cas13design.nygenome.org/) and the primer was synthesized and annealed. Stbl3 receptor cells (Weidi, Shanghai, China) were transfected with a plasmid prepared by digestion with BsmBI-v2 (New England Biolabs, Inc., Hercules, California, USA). The positive clones were selected to extract the plasmids and transfected with jetPRIME^®^ (Polyplus, Strasbourg, France) in accordance with the manufacturer’s instructions. Total RNA and protein were extracted 24 h later and verified by RT-qPCR and WB analyses, respectively. Cells transfected with empty plasmids were used as controls. The primer sequences are listed in Supplemental Table 1.

### Transfection

Targeted miRNA inhibitors and mimics were purchased from MedChemExpress (Monmouth Junction, NJ, USA). Briefly, MH-S cells were cultured with inhibitors and mimics of miR-328-5p (150 and 100 nmol/L, respectively) and miR-6975-3p (40 and 40 nmol/L, respectively) for 24 h, respectively. The same volume of solute was added as a blank control. After 24 h, the mRNA and protein levels of the target genes were measured. The ability of MH-S cells to phagocytize and kill spores was assessed as described above.

### Statistical analysis

Data analysis was conducted using GraphPad Prism software v9.0. (GraphPad Software, Inc., San Diego, CA, USA). Group comparisons were performed with the Student’s *t*-test or one-way analysis of variance. Each experiment was repeated three times. *p*-value < 0.05 was considered statistically significant.

## Results

### SNH exhibited a therapeutic effect against IPA

**Fig. 1:**
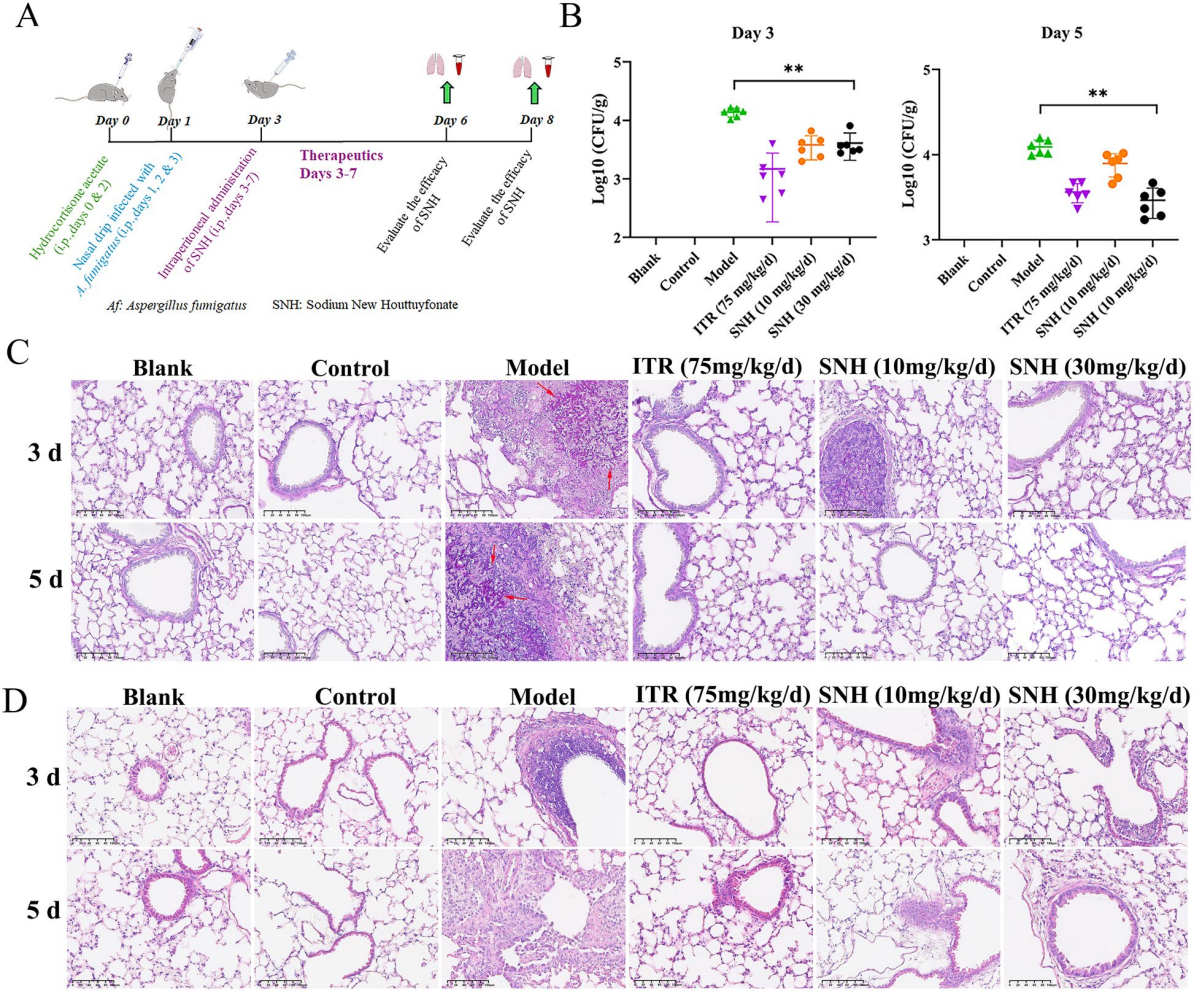
Therapeutic effect of SNH against IPA in mice. **A** A mouse model of IPA. **B** Fungal burden of the lung. The IPA model mice were treated by intraperitoneal injection with SNH at 10 and 30 mg/kg/day. The fungal burden was measured at 3 and 5 days post treatment. Histological analysis of mice with IPA. PAS-stained **C** and H&E-stained **D** sections were prepared from the lungs of mice at 3 and 5 days. Scale bar = 100 µm. *p < 0.05, **p < 0.01 *vs*. the model group.

The therapeutic effect of SNH was evaluated by constructing a mouse model of IPA through nasal drip infection (Fig. 1A). After 3 and 5 days of administration, the fungal load in the lung tissues of mice treated with SNH at 10 and 30 mg/kg/day was significantly reduced as compared to the model group (*p* < 0.05) (Fig. 1B). PAS staining showed branching of *A. fumigatus* in the lung tissues of untreated mice (Fig. 1C). In addition, H&E staining showed that as compared to the untreated group, the inflammatory cell infiltration of the lung tissues was reduced in the SNH treated group, the alveolar structure was relatively intact, and the bronchiole wall structure was relatively clear (Fig. 1D), suggesting a therapeutic effect of SNH.

**Fig. 2:**
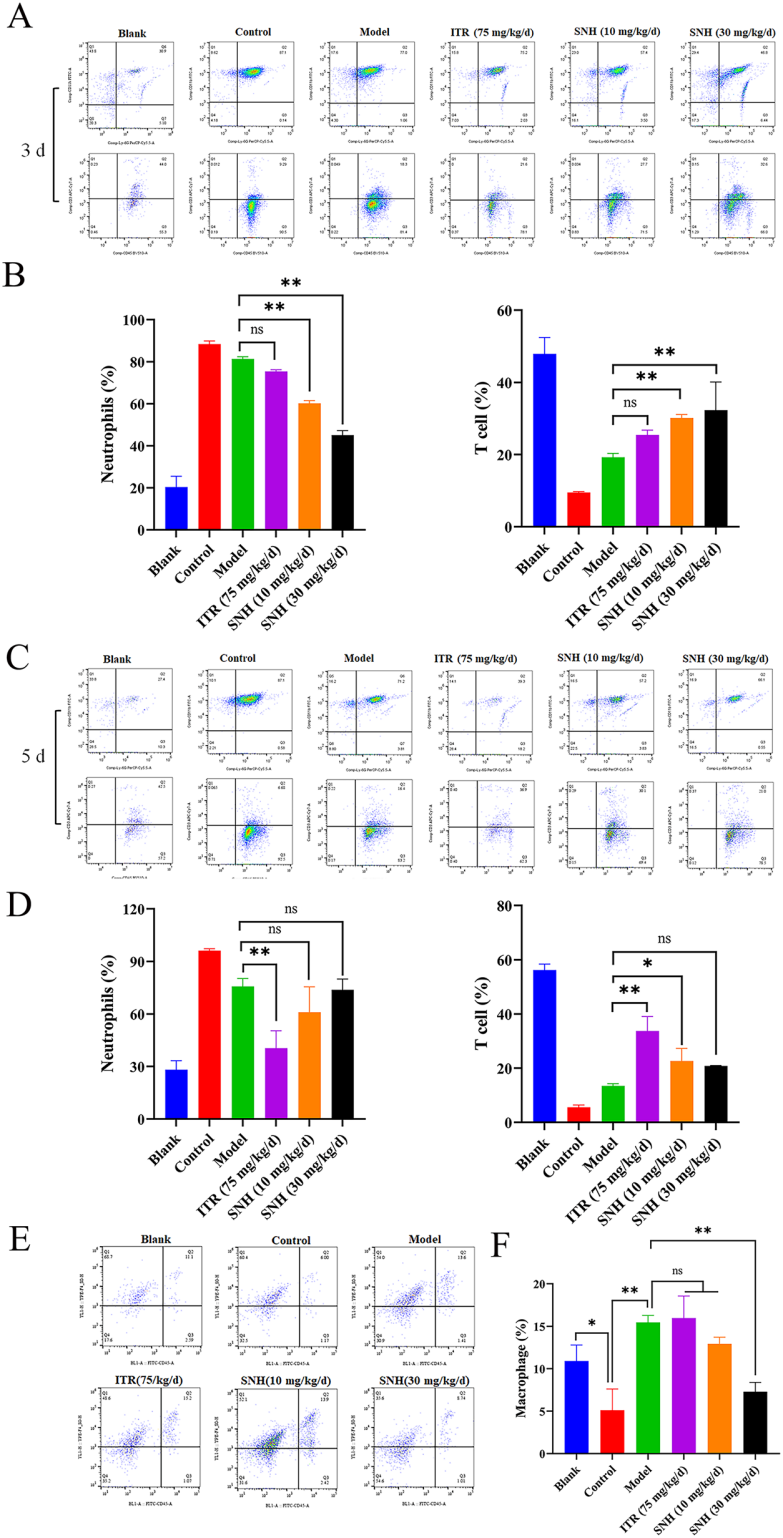
Detection of immune cells in IPA mice. The proportions of neutrophils and T cells in the lungs of mice after 3 (**A**, **B**) and 5 (**C**, **D**) days of SNH treatment. The proportion of AMs in the lungs of mice after 3 days (**E**, **F**) of SNH treatment. *p < 0.05 and **p < 0.01 , *ns*= not significant *vs*. the model or control group.

Infiltration of neutrophils and T cells at the site of infection is suggestive of *A. fumigatus* infection [15, 18]. As compared to the model group, administration of SNH for 3 days significantly decreased the proportion of neutrophils in the lung tissues of IPA mice (Figs. 2A and B). After 5 days of administration, there was no significant difference in the proportion of neutrophils between the SNH and model groups (Figs. 2C and D). Meanwhile, administration SNH for 3 days significantly increased the proportion of T cells (Figs. 2A and B). However, administration of SNH at 10 mg/kg/d for 5 days significantly increased the proportion of T cells (Figs. 2C and D).

AMs account for more than 90% of the lung leukocytosis and are the first line of defense against infection [8]. Infection with *A. fumigatus* significantly increased the proportion of AMs in the lung tissue of mice in the model group as compared to the blank and control groups (Fig. 2E and F).

### SNH enhanced the ability of MH-S cells to phagocytize and kill *A. fumigatus* conidia

**Fig. 3:**
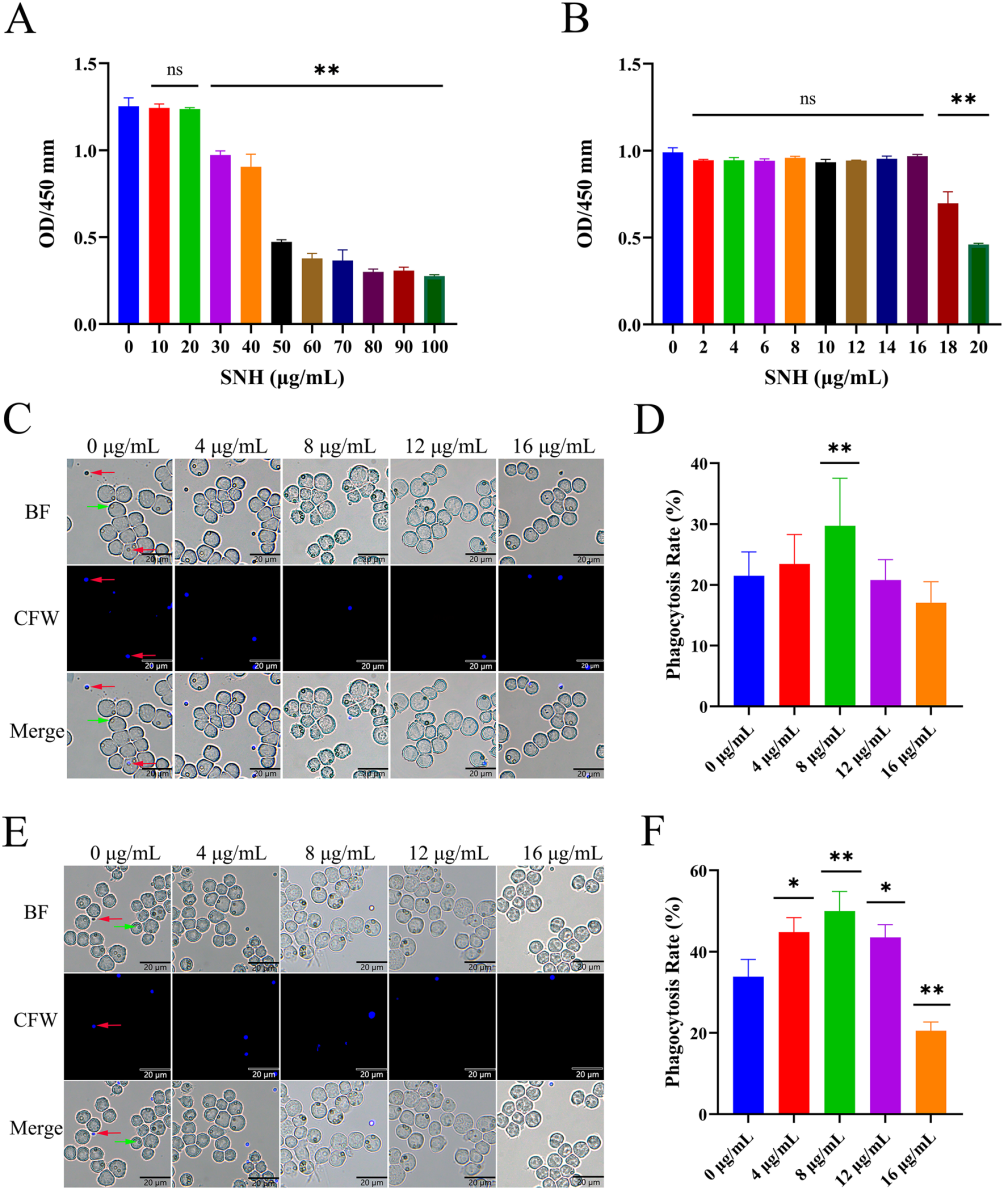
Cytotoxicity of SNH and phagocytosis ability of MH-S cells. The vitality of MH-S cells after co-culture with SNH for 2 h (**A**) and 4 h (**B**). MH-S cells were cultured with SNH for 2 h (**C**, **D**) and 4 h (**E**, **F**), followed by the addition of *A. fumigatus* conidia at an MOI of 5, then MH-S cells was observed with a fluorescence microscope, the green arrow points to phagocytic conidia, the red arrow points to unphagocytic conidia. Scale bar = 20 μm. BF: bright field; CFW, calcium fluorescent white stain. *ns =* not significant. *p < 0.05 and **p < 0.01 *vs*. MH-S cells treated with SNH at 0 μg/mL.

More than 90% of MH-S cells were viable after treatment with SNH at 16 μg/mL for 2 h and 20 μg/mL for 4 h (Fig. 3A, B). Moreover, SNH at 16 μg/mL had no significant effect on the morphology of MH-S cells (Supplementary Fig. 1), thus below this concentration was used for further investigations.

To determine whether SNH affected phagocytosis, MH-S cells were incubated with SNH for 2 h and then infected with *A. fumigatus* for another 2 h. Endocytosis of conidia by MH-S cells was significantly increased after culture with SNH for 2 and 4 h (Figs. 3C–F). In addition, the effect of SNH on the ability of MH-S cells to kill the conidia was determined by counting the number of CFUs. The results showed SNH at 8 μg/mL for 2 h had no significant effect on the ability of MH-S cells to kill conidia and was significantly enhanced after 4 h (Supplementary Fig. 2). Therefore, SNH at 8 μg/mL was used in the following experiment.

The production of ROS plays an important role in the clearance of pathogenic fungi [50]. However, SNH had no significant effect on production of ROS by MH-S cells (Supplementary Fig. 3), while ROS production was decreased in MH-S cells infected with *A. fumigatus* conidia (Supplementary Fig. 4).

### SNH activated the TLR2/TRAF6/IRF5 pathway in MH-S cells

**Fig. 4:**
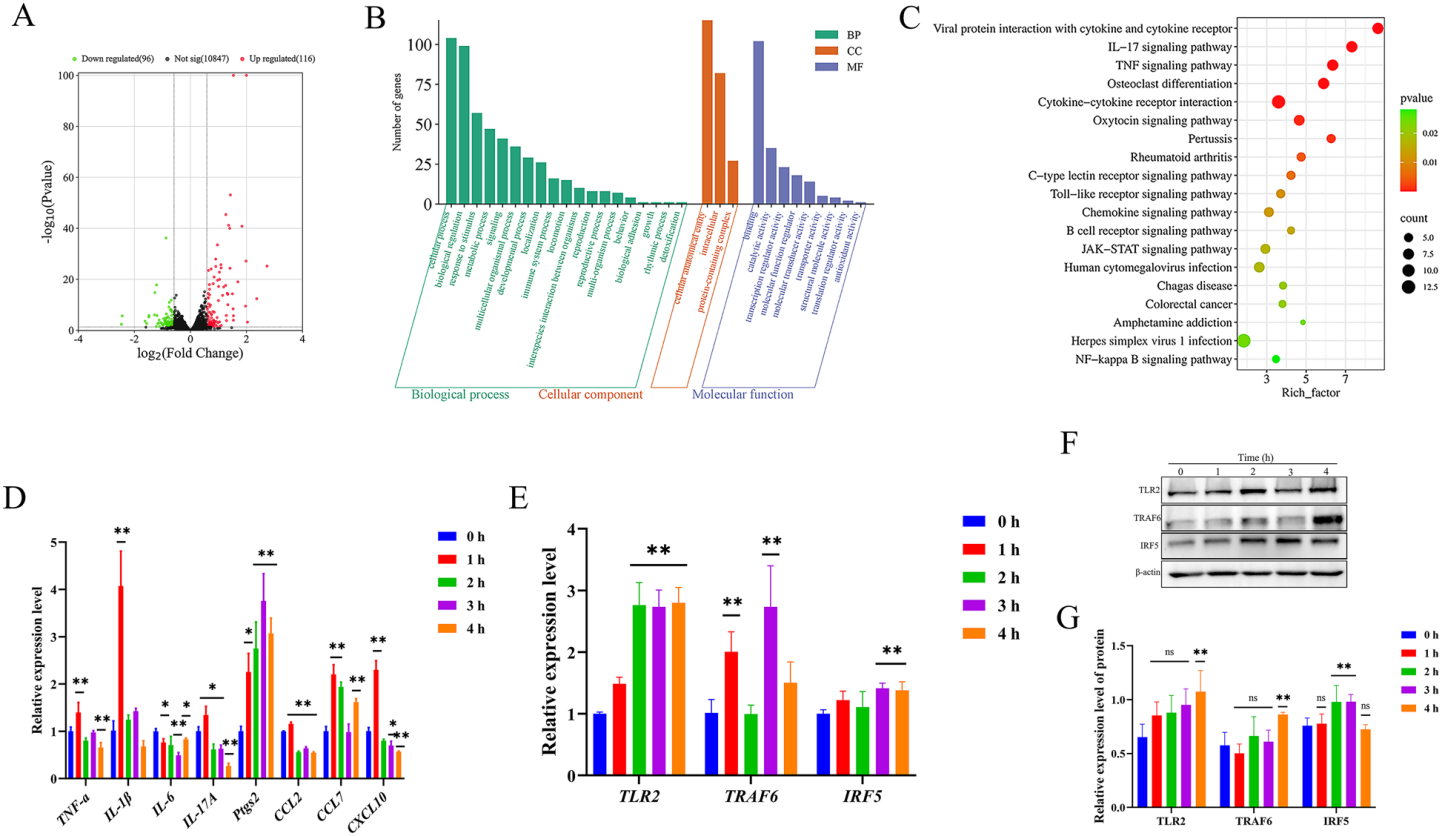
SNH activated the TLR2/TRAF6/IRF5 pathway to regulate expression of genes related to inflammation and chemokine production in MH-S cells. MH-S cells were cultured with SNH at 37 °C for 1 h under an atmosphere of 5% CO_2_. Volcanic map (**A**), GO analysis (**B**), and KEGG pathways (**C**) of DEGs. Expression levels of genes related to inflammation and chemokine production (**D**). The expression levels of signaling pathway-related genes (**E**) and proteins (**F**, **G**) were changed in MH-S cells cultured with SNH at 37 °C under an atmosphere of 5% CO_2_ at 0, 1, 2, 3, and 4 h. *ns=* not significant. *p < 0.05 and **p < 0.01 *vs*. MH-S cells co-cultured with SNH for 0 h.

Transcriptome analysis was performed to clarify the potential regulatory mechanism of SNH to regulate the ability of MH-S cells to phagocytize and kill *A. fumigatus* conidia. In non-infected MH-S cells, 10,847 transcripts and 212 DEGs (116 up-regulated and 96 down-regulated) were identified (Fig. 4A). GO analysis showed that the DEGs were mainly enriched in 31 items, with biological process as the most enriched with 19 items. The top there items were cellular process (104), biological regulation (99), and response to stimulus (57). Molecular function was second with nine items, while cellular component accounted for only three items (Fig. 4B). KEGG enrichment analysis showed enrichment of DEGs in TLR signaling pathways (Fig. 4C). The DEGs Jun proto-oncogene (*Jun*), macrophage inflammatory protein (*MIP*)*-1α*, and *MIP-1β* were up-regulated by 0.64, 1.36, and 1.09 fold, respectively, while expression of sterile alpha and TIR motif containing 1 (*SARM1*), which causes cell degeneration and death by overactivation, was down-regulated by 0.78 fold.

RT-qPCR analysis was employed to measure the expression levels of genes coding for TLRs, interleukin (IL)-17, and tumor necrosis factor (TNF) receptors in MH-S cells co-cultured with SNH. The results showed that the transcript levels of *TNF-α*, IL*-1β*, *IL-17A*, and chemokine (C–C motif) ligand 2 (*CCL2*), C-X-C motif chemokine ligand 10 (*CXCL10*) were up-regulated at 1 h and then down-regulated at 2–4 h, while the transcript levels of *CCL7* and prostaglandin-endoperoxide synthase 2 (*Ptgs2*) were up-regulated and *IL-6* was down-regulated with prolonged culture of 1–4 h (Fig. 4D). Further, the RT-qPCR and WB assays were used to clarify the biological mechanisms regulating changes to gene expression levels in treated MH-S cells. The results showed that SNH increased the expression levels of *TLR2* and *IRF5* with time in MH-S cells, while mRNA expression of TRAF6 was increased at 1 and 3 h (Fig. 4E). Also, the protein expression levels of TLR2 and TRAF6 were increased at 4 h, while protein expression of IRF5 was increased at 2 and 3 h (Fig. 4F, G). These findings suggest that SNH promoted activation of the TLR2/TRAF6/IRF5 axis in MH-S cells.

### SNH activated the TLR2/TRAF6/ERK pathway in MH-S cells infected with *A. fumigatus* conidia

**Fig. 5:**
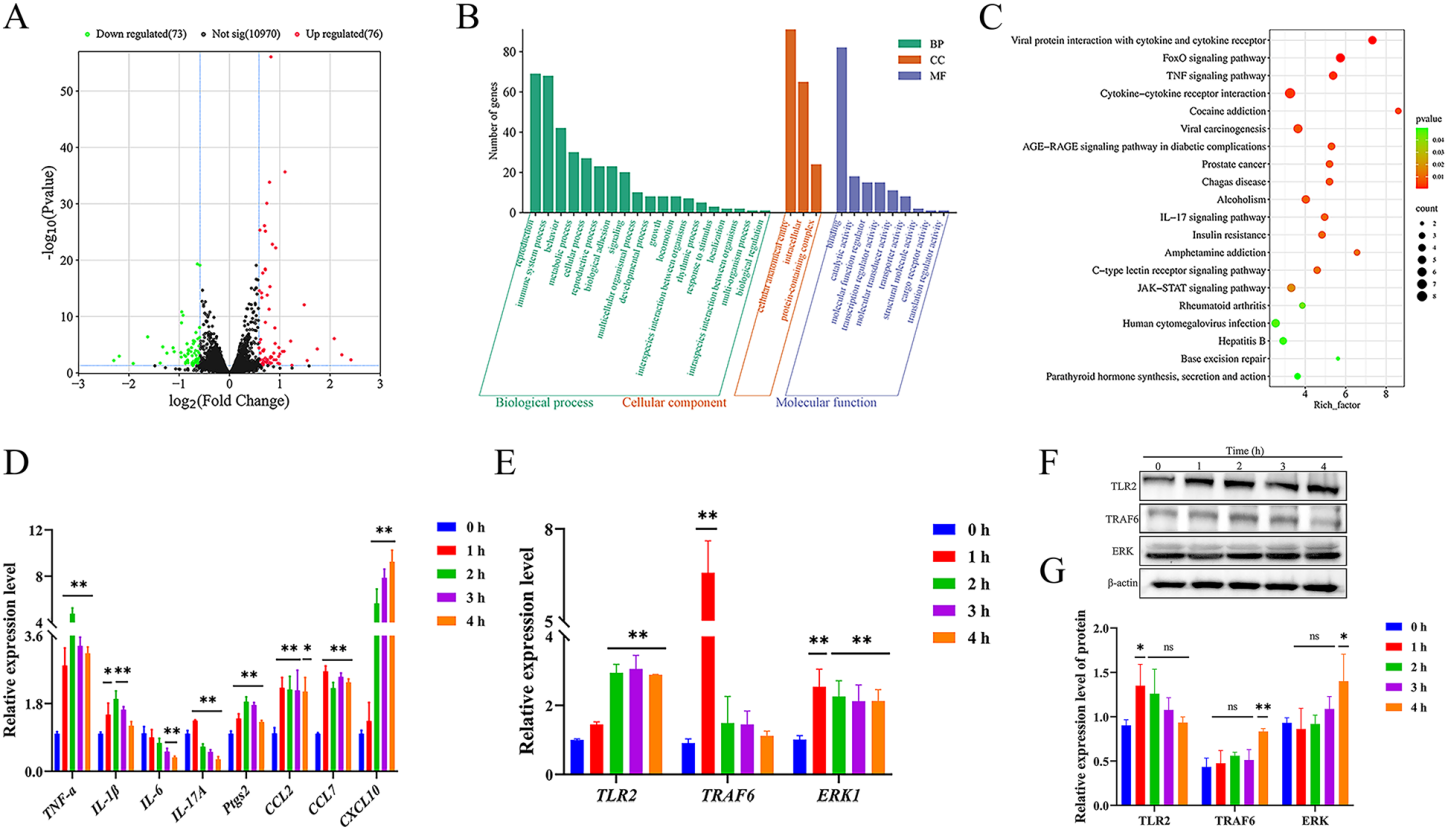
SNH activated the TLR2/TRAF6/ERK1 pathway to regulate the expression of genes related to inflammation and chemokines production in MH-S cells infected with *A. fumigatus* conidia. MH-S cells infected with *A*. *fumigatus* conidia for 2 h were cultured with SNH at 37 °C for 1 h under an atmosphere of 5% CO_2_. Volcanic map (**A**), GO analysis (**B**), and KEGG pathways (**C**) of DEGs. Expression levels of genes related to inflammation and chemokine production (**D**). Changes to the expression levels of signaling pathway-related genes (**E**) and proteins (**F**, **G**) in MH-S cells infected with *A. fumigatus* conidia and cultured with SNH at 37 °C under an atmosphere of 5% CO_2_ at 0, 1, 2, 3, and 4 h. *ns=* not significant. *p < 0.05 and **p < 0.01 *vs*. MH-S cells infected with *A. fumigatus* conidia and cultured with SNH for 0 h.

In MH-S cells infected with *A. fumigatus* conidia, 10,970 transcripts and 149 DEGs (76 up-regulated and 73 down-regulated) were identified (Fig. 5A). GO analysis showed that DEGs were mainly enriched in 31 items, with biological process as the most enriched with 19 items. The top three items were cellular process (69), immune system process (68), and behavior (42). Molecular function was second with nine items, while cellular component accounted for only three items (Fig. 5B). KEGG enrichment analysis showed enrichment of DEGs in cytokine-cytokine receptor interaction signaling pathways (Fig. 5C). The expression levels of the DEGs *Jun*, *MIP-1α*, and *MIP-1β* were up-regulated by 0.64, 0.76, and 0.85 fold, respectively.

RT-qPCR analysis also was used to measure the expression levels of genes related to TNF receptors, cytokine-cytokine receptor interactions, and IL-17 signaling pathways in MH-S cells infected with *A. fumigatus* conidia and co-cultured with SNH. The results showed that *IL-17A* was up-regulated at 1 h and then down-regulated at 2–4 h, while transcript levels of *TNF-α*, *IL-1β*, *Ptgs2*, *CCL2*, *CCL7*, and *CXCL10* were up-regulated and *IL-6* was down-regulated with prolonged culture of 1–4 h (Fig. 5D). Further RT-qPCR and western blot assays were used to clarify the biological mechanisms regulating changes to gene expression levels in MH-S cells infected with *A. fumigatus* conidia. The results showed that SNH increased expression of *TLR2* and *ERK1* with time in MH-S cells infected with *A. fumigatus* conidia, while mRNA expression of *TRAF6* was increased at 1 h (Fig. 5E). Meanwhile, protein expression of TLR2 was increased at 1 h and TRAF6 and ERK were increased at 4 h (Fig. 5F, G). These findings suggest that SNH promoted activation of the TLR2/TRAF6/ERK axis in MH-S cells infected with *A. fumigatus* conidia.

### SNH and TLR2 exhibited a combined anti-*A. fumigatus* effect

**Fig. 6:**
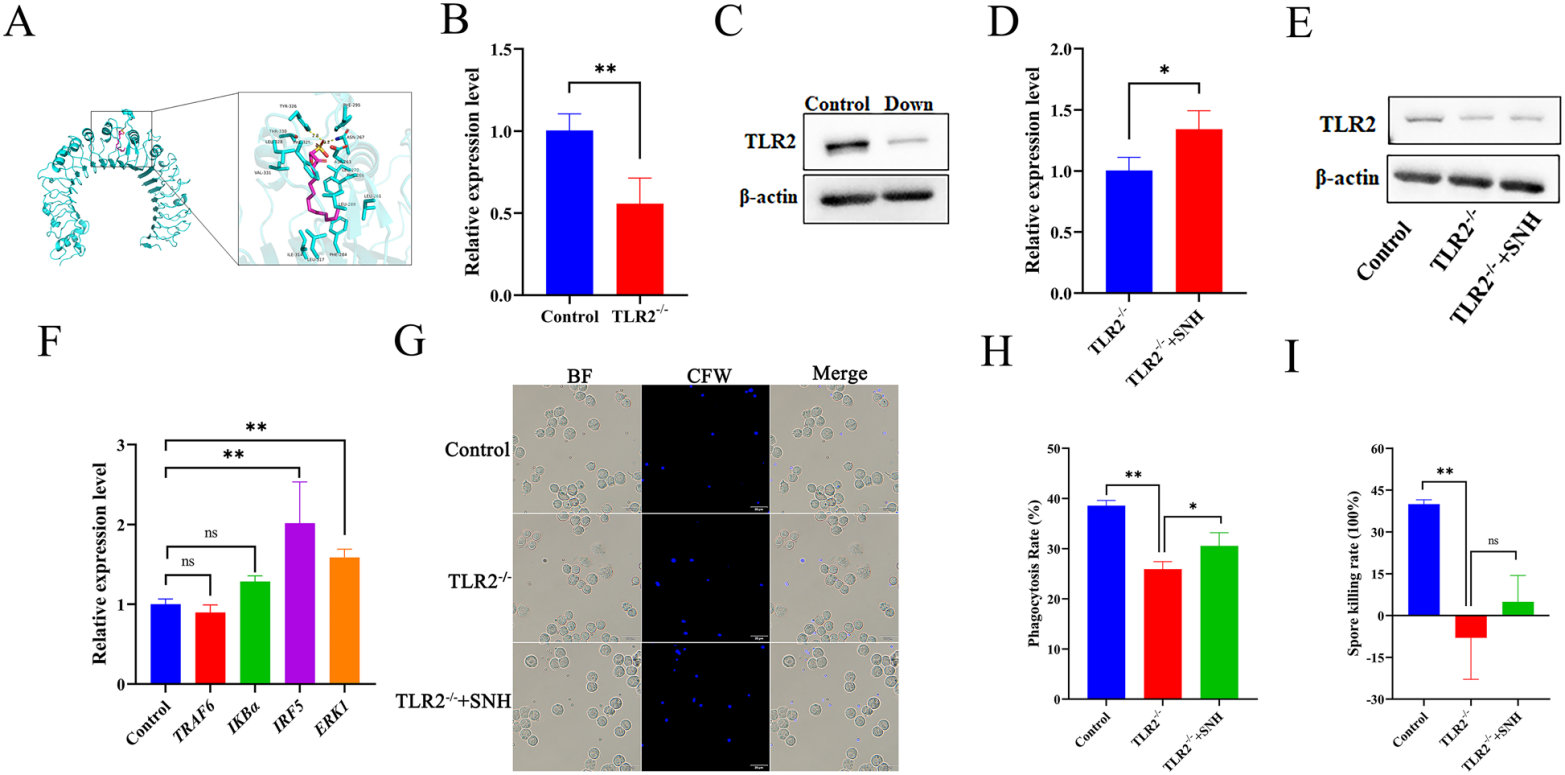
SNH activated the IRF5- and ERK1-related pathways to regulate the phagocytosis ability of MH-S after recognition by TLR2. **A** Molecular docking analysis of SNH and TLR2 receptor (PDBID: 2z81) in mice. TLR2-knockdown MH-S cells were constructed (TLR2^−/−^). The mRNA (**B**) and protein (**C**) expression levels of TLR2 in TLR2^−/−^ cells *vs*. control. Compensation effect of SNH on the mRNA (**D**) and protein (**E**) expression levels of TLR2 in TLR2^−/−^ cells. The effect of SNH on the mRNA expression levels of *IRF5* and *ERK1* (**F**), and the phagocytosis and killing abilities of TLR2^−/−^ cells (**G**, **I**). BF: bright field; CFW, calcium fluorescent white stain. *ns=* not significant. *p < 0.05 and **p < 0.01 *vs*. MH-S cells treated with SNH at 0 h.

To clarify that whether SNH directly act with TLR2, ChimeraX software was used to determine the interaction targets. The results showed that SNH docked with TLR2, with a binding energy of -6.54 kCal/mol, and the phosphate head of SNH formed hydrogen bonds with the receptor via a TYR residue at position 326 and an ASN residue at position 267 (Fig. 6A).

To further clarify the direct relationship between SNH and TLR2, *TLR2* gene expression was inhibited by CRISPR-Cas 13d technology. The RT-qPCR and WB results showed that TLR2-knockdown cells were successfully constructed (TLR2^−/−^) (Fig. 6B, C). Meanwhile, TLR2^−/−^ cells cultured with SNH for 4 h could partially compensate the mRNA and protein expression levels of TLR2, while the transcript levels of *IRF5* and *ERK1* were up-regulated (Fig. 6D, E, F). In addition, staining with calcium fluorescent white and plate CFUs counts showed that as compared to the control group, the ability of TLR2^−/−^ cells to phagocytize and kill *A. fumigatus* conidia was significantly reduced, which was compensated by incubation with SNH for 4 h (Fig. 6G, H, I). These results indicate that binding of SNH to TLR2 on the macrophage membrane had promoted activation of the TRAF6/IRF5 and TRAF6/ERK1 pathways in MH-S cells regardless of infection with *A. fumigatus* conidia.

### SNH inhibited expression of miR-328-5p and miR-6975-3p in MH-S cells

**Fig. 7:**
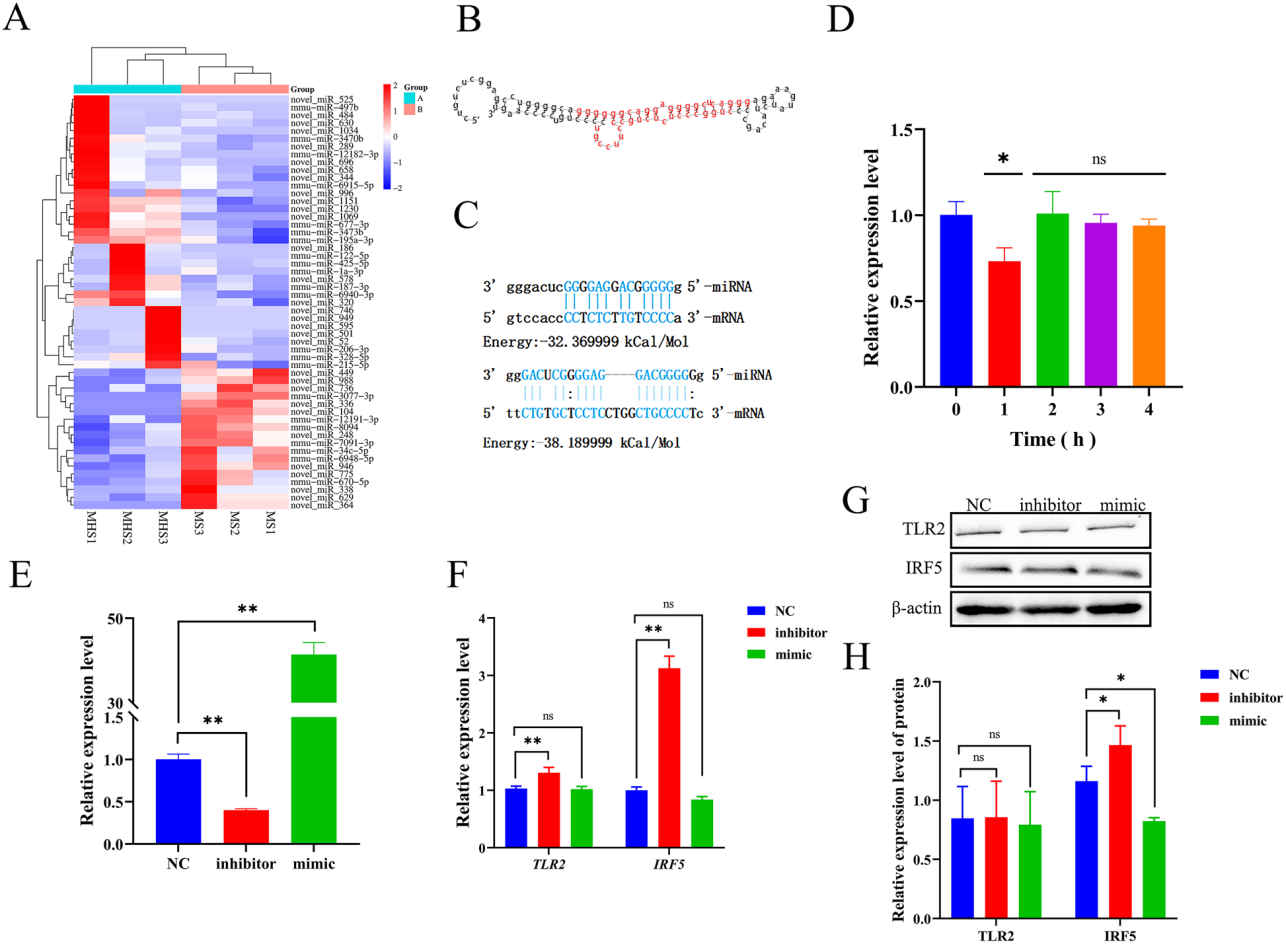
SNH regulates miRNA expression in MH-S cells. MH-S cells were cultured with SNH at 37 °C for 1 h under an atmosphere of 5% CO_2_. A heat map of miRNA expression (**A**). The sequence of miR-328-5p (**B**) and binding sites for TLR2 and IRF5 (**C**). Expression of miR-328-5p was down-regulated in MH-S cells cultured with SNH at 37 °C for 1 h under an atmosphere of 5% CO_2_ (**D**). MH-S cells were cultured with the miR-328-5p inhibitor or mimic at 37 °C for 24 h under an atmosphere of 5% CO_2_. Expressions levels of miR-328-5p (**E**) and the mRNA (**F**) and protein (**G**, **H**) levels of TLR2 and IRF5. MHS, mouse AMs; MS, mouse AMs cultured with SNH; NC: MH-S cells cultured without the inhibitor or mimics. *ns=* not significant. *p < 0.05 and **p < 0.01 *vs*. MH-S cells cultured with SNH for 0 h or without inhibitors and mimics.

**Fig. 8:**
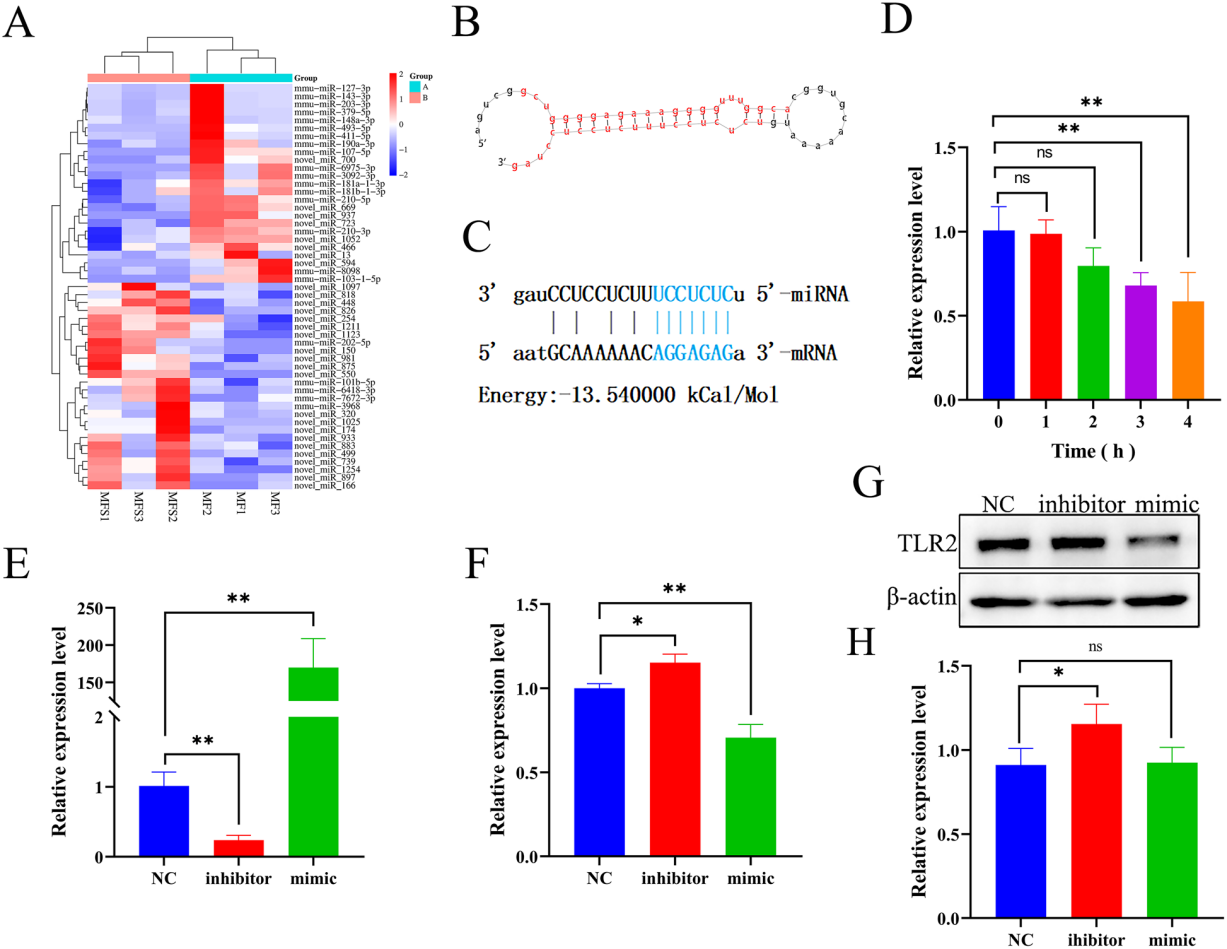
SNH regulates miRNA expression in MH-S cells infected with *A. fumigatus* conidia. MH-S cells were cultured with SNH at 37 °C for 1 h under an atmosphere of 5% CO_2_. A heat map of miRNA expression (**A**). The sequence of miR-6975-3p (**B**) and binding sites for TLR2 (**C**). Expression of miR-6975-3p was down-regulated in MH-S cells infected with *A. fumigatus* conidia and cultured with SNH at 37 °C for 3 and 4 h under an atmosphere of 5% CO_2_ (**D**). MH-S cells were cultured with the miR-6975-3p inhibitor or mimic at 37 °C for 24 h under an atmosphere of 5% CO_2_. Expressions levels of miR-6975-3p (**E**) and the mRNA (**F**) and protein (**G**, **H**) levels of TLR2. MF, mouse AMs infected with *A. fumigatus* conidia; MFS, mouse AMs infected with *A. fumigatus* conidia cultured with SNH; NC: MH-S cells cultured without the inhibitor or mimics. *ns=* not significant. *p < 0.05 and **p < 0.01 *vs*. MH-S cells cultured with SNH for 0 h or without inhibitors and mimics.

MiRNAs are important regulators of phenotypic reprogramming of macrophages in response to inflammatory stimulation [47, 51]. Further miRNA sequencing was performed to determine whether SNH has a regulatory effect on miRNA expression in MH-S cells. In non-infected MH-S cells cultured with SNH for 1 h, 53 miRNAs were differentially expressed (18 up-regulated and 35 down-regulated) (Fig. 7A). Among the differentially expressed miRNAs, expression of miR-328-5p was down-regulated by 2.56 fold. Moreover, further analysis found that miR-328-5p regulated 2024 target genes, which was far greater than the other differentially expressed miRNAs (Table 1). Further miRNA_mRNA target gene interaction analysis found that miR-328-5p had an incomplete binding site with the coding regions of TLR2 and IRF5, with binding energies of − 32.36999 and − 38.1899 kCal/Mol, respectively (Figs. 7B, C). In MH-S cells infected with *A. fumigatus* conidia, as compared to untreated MH-S cells infected with *A. fumigatus* conidia, 51 miRNA were differentially expressed (26 up-regulated and 25 down-regulated) at 1 h (Fig. 8A). Among the differentially expressed miRNAs, expression of miR-6975-3p was down-regulated by 4.9559 fold. Further miRNA_mRNA target gene interaction analysis found that miR-6975-3p had an incomplete binding site with the coding region of TLR2, with a binding energy of − 13.54 kCal/Mol (Figs. 8B, C).

The results of SNH on miR-328-5p and miR-6975-3p in MH-S cells showed that miR-328-5p expression was decreased in MH-S cells cultured with SNH for 1 h (Fig. 7D). Furthermore, miR-328-5p expression was significantly decreased in MH-S cells cultured with miR-328-5p inhibitors for 24 h (Fig. 7E), while the mRNA expression levels of *TLR2* and *IRF5* were significantly increased (Fig. 7F). Moreover, the WB results showed that IRF5 expression was significantly up-regulated, while there was no significant change to TLR2 expression (Fig. 7G, H). Expression of miR-328-5p was significantly increased in MH-S cells cultured with the miR-328-5p mimics (Fig. 7E), while there was no significant change to the mRNA levels of *TLR2* and *IRF5* (Fig. 7F). At the protein level, IRF5 was significantly down-regulated, while there was no significant difference in TLR2 (Fig. 7G, H). Meanwhile, miR-6975-3p expression was decreased in MH-S cells cultured with SNH for 3 and 4 h (Fig. 8D). Furthermore, miR-6975-3p expression was significantly decreased in MH-S cells cultured with miR-6975-3p inhibitors for 24 h (Fig. 8E), and mRNA expression of *TLR2* was significantly increased (Fig. 8F). Moreover, the WB results showed that TLR2 was significantly up-regulated (Fig. 8G, H). Expression of miR-6975-3p was significantly increased in MH-S cells cultured with the miR-6975-3p mimics (Fig. 8E), while mRNA expression of *TLR2* was significantly down-regulated (Fig. 8F). At the protein level, there was no significant difference in TLR2 (Fig. 8G, H).

Fluorescence microscopy showed that phagocytosis of conidia was significantly increased in MH-S cells treated with miR-328-5p inhibitors as compared to the control group (40.54% *vs*. 50.46%, respectively). In addition, plate counts of CFUs showed that the miR-328-5p mimic significantly reduced the killing rate of MH-S cells as compared to the control group (40.90% *vs*. 15.29%, respectively). The miR-6975-3p mimic significantly reduced the killing rate of MH-S cells as compared to the control group (40.90% *vs*. 29.00%, respectively) (Supplementary Fig. 5). These results indicate that miR-328-5p and miR-6975-3p are potential targets in response to *A. fumigatus* infection.

## Discussion

IPA is mainly caused by the rapid growth of spores germinating into mycelium in the colonized lung, which results in damage to lung tissue and blockage of blood vessels [52]. At present, azoles are the first-line drugs in clinical practice for IPA. However, the azole resistance rate is as high as 55%. SNH has been clinically used for treatment of upper respiratory tract infections. And our previous study had confirmed the antifungal effect of SNH against *A. fumigatus* [35] and the safety of SNH in mice [53]. However, relatively few studies have investigated the effects of SNH on the response of AMs to *A. fumigatus* infection. In this study, the therapeutic effect and mechanisms employed by macrophages against *A. fumigatus* infection were investigated.

Under normal circumstances, after infection with *A. fumigatus*, the proportion of immune cells is increased at the infected sites. Neutrophils and T cells play important roles in the immune response against mycelia while controlling inflammation [9, 54]. In this study, therapeutic effect of SNH was linked to increased proportions of neutrophil, AMs, and T cells in the lung tissues of infected mice (Fig. 2). As we all know, macrophages play important roles in the initial recognition, clearance, and inhibition of germination of *A. fumigatus* conidia [9, 55]. In response to infection, activated macrophages at the infected site of the host promotes recruitment of neutrophils and T cells to jointly resist infection of *A. fumigatus* [2]. In addition, after infection with *A. fumigatus* conidia, the proportion of macrophages in the lungs of mice in the model group was higher than in the blank and control groups, while treatment with SNH reduced the proportion of macrophages to control inflammation (Fig. 2), which further indicated that macrophages actively respond to invasion of *A. fumigatus* conidia. Therefore, we proposed a hypothesis that the effect of SNH against IPA may be due to the combination of a lower fungal load in the lungs of IPA mice, better inflammation control, and an increased proportion of immune cells. Although SNH has a direct inhibitory effect on *A. fumigatus*, due to the complexity of its in vivo action [35], we are more inclined to believe that SNH exerts its antifungal effect by regulating the host’s immune status after entering the body. However, the specific mechanism of action still needs further exploration.

In general, the recognition, phagocytosis, and elimination of inhaled *A. fumigatus* conidia by AMs are mainly achieved through endospore injury and cytokine production. The results of the present study found that the addition of SNH to the culture medium significantly enhanced the ability of MH-S cells to phagocytize and kill conidia (Fig. 3). Meanwhile, PRR and cytokines play important roles during recognition and phagocytosis [56]. As a member of the TLR family, TLR2 was found to respond to infection by *A. fumigatus* conidia or mycelium in both mouse and human cells [57]. TRAF6, an important adaptor protein similar to Myd88, has been identified as a downstream participant in many immunomodulatory receptor families, including the TNF receptor superfamily members, TLRs, and T cell receptors [58, 59]. IRF5 is a transcription regulatory factor that plays an important role in host defense by interacting with itself and other transcription factors [60]. ERK-related signaling pathways can affect cell behavior by altering metabolic pathways and gene expression patterns. Moreover, it has been demonstrated that ERK-related signaling pathways can also play an anti-*A. fumigatus* role independently of the TLR2/4 receptors of macrophages [11, 61]. The results of the present study indicate that SNH bound directly to TLR2 and then increased the protein and mRNA expression levels of TRAF6/ IRF5 in non-infected MH-S cells (Fig. 4E–G) and TRAF6/ ERK in MH-S cells infected with *A. fumigatus* conidia (Fig. 5E-G), which could influence expression of downstream genes related to inflammation and chemokine production.

Up-regulation of *Ptgs2* and *CCL7* has been confirmed to prevent *A. fumigatus* infection by promoting phagocytosis and killing of conidia by macrophages [62]. Moreover, *Ptgs2*, a key gene of cyclooxygenase-2, is involved in the induction of protective innate immune responses against invasive aspergillosis [63]. The results of this study also confirmed that in non-infected MH-S cells, the expression levels of *Ptgs2* and *CCL7* were continuously up-regulated, and the inflammatory cytokines *TNF-α*, *IL-1β*, *IL-17A*, *CCL2*, and *CXCL10* were up-regulated in the early stage of co-culture with SNH and then down-regulated with time (Fig. 4D). However, SNH had no significant regulatory effect on *NF-κB* expression in MH-S cells (Supplementary Fig. 6). These findings suggest that these changes may be closely related to expression of *p38* mitogen activated protein kinases (*p38*) in MH-S cells (Supplementary Fig. 6), suggesting that SNH-induced activation of the TLR2/TRAF6/IRF5 pathway in MH-S cells regulates expression of inflammatory factors against *A. fumigatus* infection. In MH-S cells infected with *A. fumigatus* conidia, the expression levels of *TNF-α*, *IL-1β*, *Ptgs2*, *CCL2*, *CCL7*, and *CXCL10* were continuously up-regulated (Fig. 5D), while down-regulation of the inflammatory cytokines *IL-6* and *IL-17A* may be closely related to expression of *NF-κB* and *p38* (Supplementary Fig. 7). The expression of *CCL2* and *CXCL10*, the effect to chemotactic T cells, was significantly up-regulated, which was consistent with the result that the proportion of T cells in the lungs of mice was increased after SNH treatment of IPA mice. However, the regulatory effects of SNH on T cell subsets still need to be further explored.

In cells, miRNAs specifically bind to the 3′-untranslated region (UTR) of target mRNAs to regulate expression [64]. Also, miRNAs can bind to the promoter, 5′ UTR, and coding regions of target mRNAs [65]. The functions of miRNAs in immune cells against *A. fumigatus* infection have been confirmed. For example, miR-146a plays an anti-infection role in THP-1 cells by negatively regulating secretion of TNF-α and IL-6 [66]. A prior study found that miR-132 was activated in both monocytes and dendritic cells in response to *A. fumigatus* infection [67]. However, relatively few studies have investigated the effects of miRNAs in the response of macrophages to *A. fumigatus* infection. The results of the present found that SNH inhibited expression of miR-328-5p and miR-6975-3p in both infected and non-infected MH-S cells (Fig. 7A). However, the expression levels of *TLR2* and *IRF5*, which interact with miR-328-5p, were significantly up-regulated at the late stage of co-culture (Fig. 4E). The mechanism may be due to the short miRNA recognition sequence and the interaction between miRNAs and mRNAs, which is not a one-to-one correspondence. After all, a single miRNA can often target more than one mRNA and an mRNA can also have binding sites for multiple miRNAs [68]. Therefore, further investigations are needed to confirm this mechanism.

**Fig. 9:**
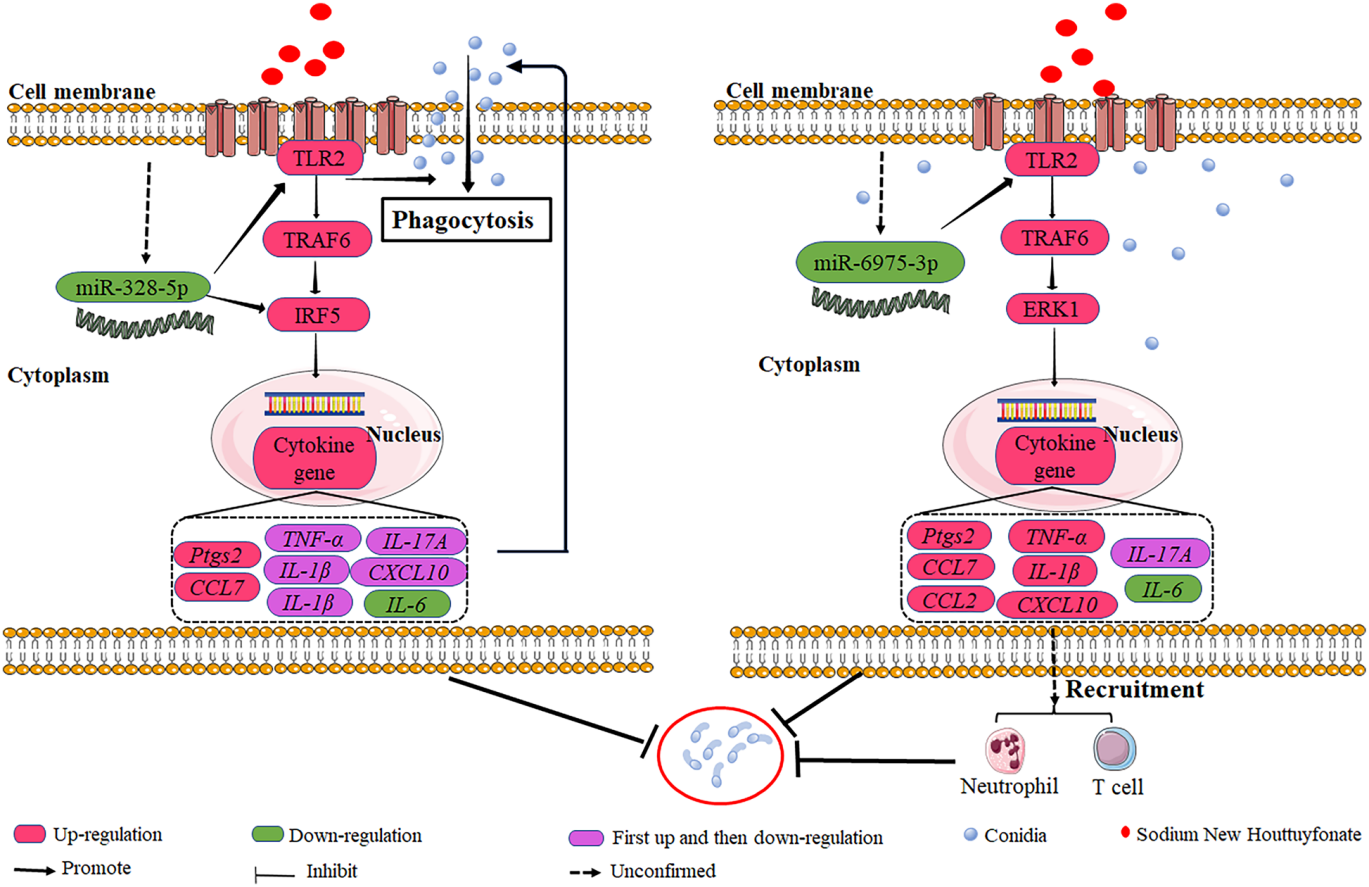
Molecular mechanism of SNH regulation in MH-S cells against *A. fumigatus* infection. SNH can directly bind to the TLR2 receptor on the cell membrane and reduce the expression of miR-328-5p and miR-6975-3p in non-infected and infected MH-S cells in non-infected and infected MH-S cells, miR-328-5p and miR-6975-3p regulated expression of genes related to inflammation and chemokine production in collaboration with the TLR2/TRAF6/IRF5 and TLR2/TRAF6/ERK1 axes. SNH promoted the ability of MH-S cells to phagocytose *A. fumigatus* conidia and recruited downstream neutrophils and T cells against *A. fumigatus* infection. Note: CCL2, chemokine (C–C motif) ligand 2,; CCL7, chemokine (C–C motif) ligand 7; CXCL10, C-X-C motif chemokine ligand 10; IL-1β, interleukin-1 beta; IL-6, interleukin-6; IL-17A, interleukin-17A; Ptgs2, prostaglandin-endoperoxide synthase 2; TNF-α, tumor necrosis factor alpha.

In summary, it confirmed that SNH plays a therapeutic role in IPA mice by reducing the fungal load in lung tissues, controlling inflammation, and increasing the proportion of neutrophils and T cells in vivo. Further mechanisms confirmed that low concentration of SNH activate TRAF6/IRF5 in non-infected MH-S cells and TRAF6/ERK in MH-S cells infected *A. fumigatus* conidia through direct interaction with TLR2 receptor. SNH also inhibited expression of miR-328-5p and miR-6975-3p, which act synergistically with the TLR2/TRAF6/IRF5 and TLR2/TRAF6/ERK axes, respectively, to exert anti-*A. fumigatus* effects, indicating that miR-328-5p/miR-6975-3p and TLR2/IRF5/ERK may be the targets of macrophages against *A. fumigatus* infection (Fig. 9). However, it must be acknowledged that this study only preliminarily elucidated the correlation between the therapeutic effect of SNH on IPA and the recruitment and proportion of macrophages, neutrophils, and T cells. Its deeper mechanism of action still needs to be explored. In addition, although this study confirmed the regulatory effects of SNH on the TLR2/TRAF6/IRF5 and TLR2/TRAF6/ERK pathways and miRNA in macrophages, further exploration is needed to determine whether SNH exerts antifungal effects by affecting miRNA expression and regulating other target mRNAs. Collectively, these results demonstrate the great potential of SNH as a candidate immunotherapy drug against *A. fumigatus* infection.

## Data Availability

The raw sequence data generated during the current study are available in the [Genome Sequence Archive of the Beijing Institute of Genomics], accession number is CRA015645. And the other datasets used during the current study are available from the corresponding author on reasonable request.

## Abbreviations

*A. fumigatus*: *Aspergillus fumigatus*
CCK-8: Cell counting kit-8
CFUs: Colony-forming units
H&E: Hematoxylin–eosin
IPA: Invasive pulmonary aspergillosis
MH-S cells: Mouse alveolar macrophages
PDA: Potato dextrose agar
PAS: Periodic acid-schiff
RT-qPCR: Real-time quantitative polymerase chain reaction
ROS: Reactive oxygen species
SNH: Sodium new houttuyfonate
TLR2: Toll like receptor 2
WB: Western blot
